# Evidence for the Sialylation of PilA, the PI-2a Pilus-Associated Adhesin of *Streptococcus agalactiae* Strain NEM316

**DOI:** 10.1371/journal.pone.0138103

**Published:** 2015-09-25

**Authors:** Eric Morello, Adeline Mallet, Yoan Konto-Ghiorghi, Thibault Chaze, Michel-Yves Mistou, Giulia Oliva, Liliana Oliveira, Anne-Marie Di Guilmi, Patrick Trieu-Cuot, Shaynoor Dramsi

**Affiliations:** 1 Institut Pasteur, Unité des Bactéries Pathogènes à Gram positif, Paris, France; 2 Centre National de la Recherche Scientifique (CNRS ERL 3526), Paris, France; 3 Institut Pasteur, Imagopole, Ultrastructural Microscopy Platform, Paris, France; 4 Institut Pasteur, Spectrométrie de Masse Structurale et Protéomique, Paris, France; 5 INRA UMR 1319, MICALIS, Jouy-en-Josas, France; 6 Université Grenoble Alpes, Institut de Biologie Structurale (IBS), Grenoble, France; University of Texas-Houston Medical School, UNITED STATES

## Abstract

*Streptococcus agalactiae* (or Group B Streptococcus, GBS) is a commensal bacterium present in the intestinal and urinary tracts of approximately 30% of humans. We and others previously showed that the PI-2a pilus polymers, made of the backbone pilin PilB, the tip adhesin PilA and the cell wall anchor protein PilC, promote adhesion to host epithelia and biofilm formation. Affinity-purified PI-2a pili from GBS strain NEM316 were recognized by *N*-acetylneuraminic acid (NeuNAc, also known as sialic acid) specific lectins such as *Elderberry Bark* Lectin (EBL) suggesting that pili are sialylated. Glycan profiling with twenty different lectins combined with monosaccharide composition by HPLC suggested that affinity-purified PI-2a pili are modified by *N*-glycosylation and decorated with sialic acid attached to terminal galactose. Analysis of various relevant mutants in the PI-2a pilus operon by flow-cytometry and electron microscopy analyses pointed to PilA as the pilus subunit modified by glycosylation. Double labeling using PilB antibody and EBL lectin, which specifically recognizes *N*-acetylneuraminic acid attached to galactose in **α**-2, 6, revealed a characteristic binding of EBL at the tip of the pilus structures, highly reminiscent of PilA localization. Expression of a secreted form of PilA using an inducible promoter showed that this recombinant PilA binds specifically to EBL lectin when produced in the native GBS context. *In silico* search for potentially glycosylated asparagine residues in PilA sequence pointed to N427 and N597, which appear conserved and exposed in the close homolog RrgA from *S*. *pneumoniae*, as likely candidates. Conversion of these two asparagyl residues to glutamyl resulted in a higher instability of PilA. Our results provide the first evidence that the tip PilA adhesin can be glycosylated, and suggest that this modification is critical for PilA stability and may potentially influence interactions with the host.

## Introduction

The Gram-positive bacterium *Streptococcus agalactiae* (also known as Group B *Streptococcus* or GBS) is the leading cause of neonatal infections and an emerging cause of invasive infections in the elderly and adults with underlying diseases [1, 2]. GBS asymptomatically colonizes the intestinal and urogenital mucosal surfaces of 30% of healthy persons [3]. Therefore, GBS is considered a human-adapted commensal bacterium, which can turn into a life-threatening pathogen under certain circumstances.

Surface proteins are known to play important roles in host-bacteria interplays, especially during adhesion and colonization. A number of adhesins have been identified and characterized in GBS [4]. Our functional analysis of the first pilus locus characterized in GBS, the PI-2a pilus in strain NEM316, showed that it encodes a major pilin (PilB) and two minor pilins subunits (PilA and PilC) that are covalently polymerized by the action of two enzymes belonging to the sortase C family [5]. One accessory pilin, PilA, is responsible for the adhesive property of the pilus [6] whereas the second, PilC, is the base subunit allowing anchoring of the polymer to the cell wall [7]. Srr1 is another cell wall anchored protein playing a key role in GBS pathogenesis [8], [9]. Srr1 is a serine-rich glycosylated protein, secreted by the accessory SecA2 pathway, whose *O-*glycosylation profile has been explored thoroughly [10]. Serendipitously, we found that Rga, a RofA-like member, whose gene is located upstream from *srr1*, is the master regulator of both Srr1 and PI-2a pilus in strain NEM316 [11]. The co-regulation of *srr1* and pili by Rga led us to question the glycosylation status of pili.

Protein glycosylation, the addition of a sugar moiety on a protein backbone, is a common post-translational modification that was considered to be restricted to eukaryotes. This dogma is no longer true and it is now firmly established that protein glycosylation takes place in Bacteria and Archaea [12]. The first bacterial glycosylated proteins that were identified are abundant surface proteins like flagellins, pilins, and S-layer proteins [13]. In the last decade, there have been increasing reports on protein glycosylation systems in Gram-negative bacteria, particularly amongst mucosal-associated pathogens like *Campylobacter jejuni* and *Neisseria spp*., that led to the genetic and biochemical characterization of *O*- and *N*-linked glycosylation systems in these two species (for a review [14]).

The role of glycans in protein function across kingdoms is mostly unknown. Nevertheless, it is assumed that this post-translational modification provide labels for binding and recognition by other proteins and/or stabilize proteins by increasing their thermodynamic stability, solubility, or resistance to proteases.

Here we investigated the glycosylation status of PI-2a pilus in GBS strain NEM316 using lectin profiling and dosage of monosaccharides on affinity purified pili. Further experiments using flow-cytometry, immunogold labeling and western blot analyses strongly suggest that PilA, the pilus-associated adhesin, displays sialic acid carbohydrate attached to terminal galactose by a **α**-2, 6 linkage. Sialylation of both capsular polysaccharide and surface proteins in GBS might represent a molecular mimicry mechanism to efficiently evade host immune defenses.

## Results

### Purified PI-2a pili are recognized specifically by the EBL (or SNA) lectin

**Fig 1:**
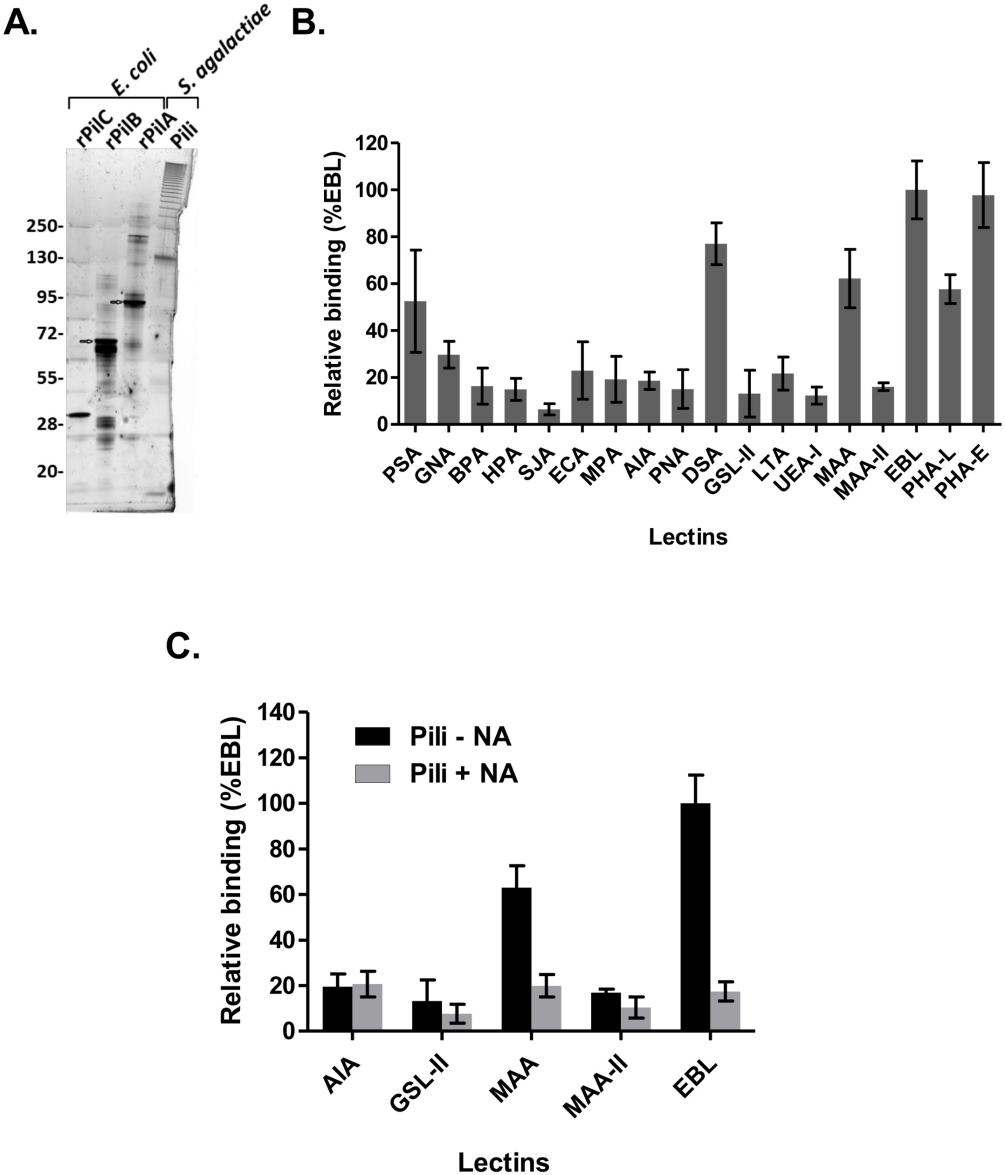
Interaction of *S*. *agalactiae* NEM316 purified pili with specific lectins. (A) Affinity purified PI-2a pili isolated from cell-wall extracts of *S*. *agalactiae* NEM316 were visualized by SDS-PAGE and silver staining analysis. His-tagged pilus subunits PilA, PilB and PilC produced in *E*. *coli* were loaded as controls. The typical ladder-like pattern corresponding to different sized of pili oligomers was obtained with purified pili sample, contamination by any other proteins could not be detected. (B) Affinity purified pili interaction profile obtained with 18 lectins displaying different carbohydrate affinities (see S1 Table). Biotinylated pili were incubated with lectins immobilized on microtiter plates in duplicate. Interaction was revealed with streptavidin-conjugated peroxidase. (C) Affinity purified pili interaction profile obtained with sialic acid specific lectins (MAA, MAA-II and EBL) after treatment (+NA) or not (-NA) of pili with neuraminidase. Values are mean ± SD that were normalized using signals measured with the most efficient binding lectin (EBL) as reference. For each value, the background signal obtained without any immobilized lectin was subtracted. The assay was performed in duplicate and repeated at least two times independently.

To test whether PI-2a pili from GBS strain NEM316 were glycosylated, we first purified the pili polymers from mutanolysin-treated cell wall extracts of the wild type strain NEM316 by immuno-affinity chromatography with polyclonal antibodies against PilB, the major pilin. Purified pili analyzed by silver stained SDS-PAGE exhibit the typical laddering pattern of covalent polymers (Fig 1A). Since mutanolysin-treated extracts also contain major surface polysaccharides such as capsule that could interfere with the analysis of pilus glycosylation, it was important to test the purity of our purified PI-2a fraction. As shown in Fig A in S1 Fig, the PI-2A fraction did not react with the antibody against type III capsular polysaccharide but did show positive signal with antibodies against the three PilA, PilB and PilC pilus subunits. The specificity of the antibodies against type III capsule and against PilA is also shown (Fig B in S1 Fig). The glycosylation profile of affinity-purified PI-2a pili was carried out by incubating biotinylated pili with eighteen lectins immobilized on 96 wells plates (Fig 1B and S1 Table). The data suggest that PI-2a pili contained carbohydrates such as glucose (PSA), *N-*acetylglucosamine (DSA), mannose (PSA, GNA) and sialic acid (MAA, EBL). Complex carbohydrates structures (Galactose β1–4 *N*-acetylglucosamine β1–2 or β1–6 mannose of bi-and tri-antennary) recognized by the lectins PHA-L and PHA-E were also detected (Fig 1B). We decided to focus on sialic acid modifications.

Specific interaction of purified pili and EBL lectin was confirmed in a reciprocal experiment in which biotinylated EBL lectin was incubated with purified pili immobilized on a 96 wells plate (S3 Fig). Treatment of pili with neuraminidase strongly reduced the signal detected with the MAA and EBL lectins to the background level (Fig 1C) whereas AIA and GSL-II lectins recognizing galactose and terminal N-acetylglucosamine residues, respectively, were found insensitive to neuraminidase activity (Fig 1C). These results further demonstrate that EBL and MAA lectins are specific for sialic acid in a terminal position.

Independently, HPLC dosage of six monosaccharides (fucose, galactosamine, glucosamine, glucose, galactose, mannose) and three different sialic acids (*N*-acetylneuraminic-, *N*-glycolylneuraminic-, 3-deoxy-d-glycero-d-galacto-2-nonulosonic (KDN)) confirmed the presence of glucose, glucosamine, galactose, mannose and *N-*acetylneuraminic acid groups on purified PI-2a pili (Table 1).

**Table 1:** HPAEC quantification of monosaccharide and sialic acid released after acid hydrolysis of affinity purified PI-2a pili from *S*. *agalactiae* strain NEM316.

Carbohydrate	Quantity (nmoles)*
**Monosaccharides** (50 μg of PI-2a injected)	
Fucose	ND**
Galactosamine	ND
Galactose	3.767 ± 0.2597
Glucosamine	8.170 ± 1.3130
Glucose	11.690 ± 1.0370
Mannose	5.147 ± 0.3516
**Sialic acid** (25 μg of PI-2a injected)	
*N*-acetylneuraminic acid	4.35
*N*-glycolylneuraminic	ND
KDN	ND

*Mean ± SD

** Not detected

Limit of detection: 0.1 μg/mL for each monosaccharide.

### The EBL lectin binds to the PilA subunit of PI-2a

**Fig 2:**
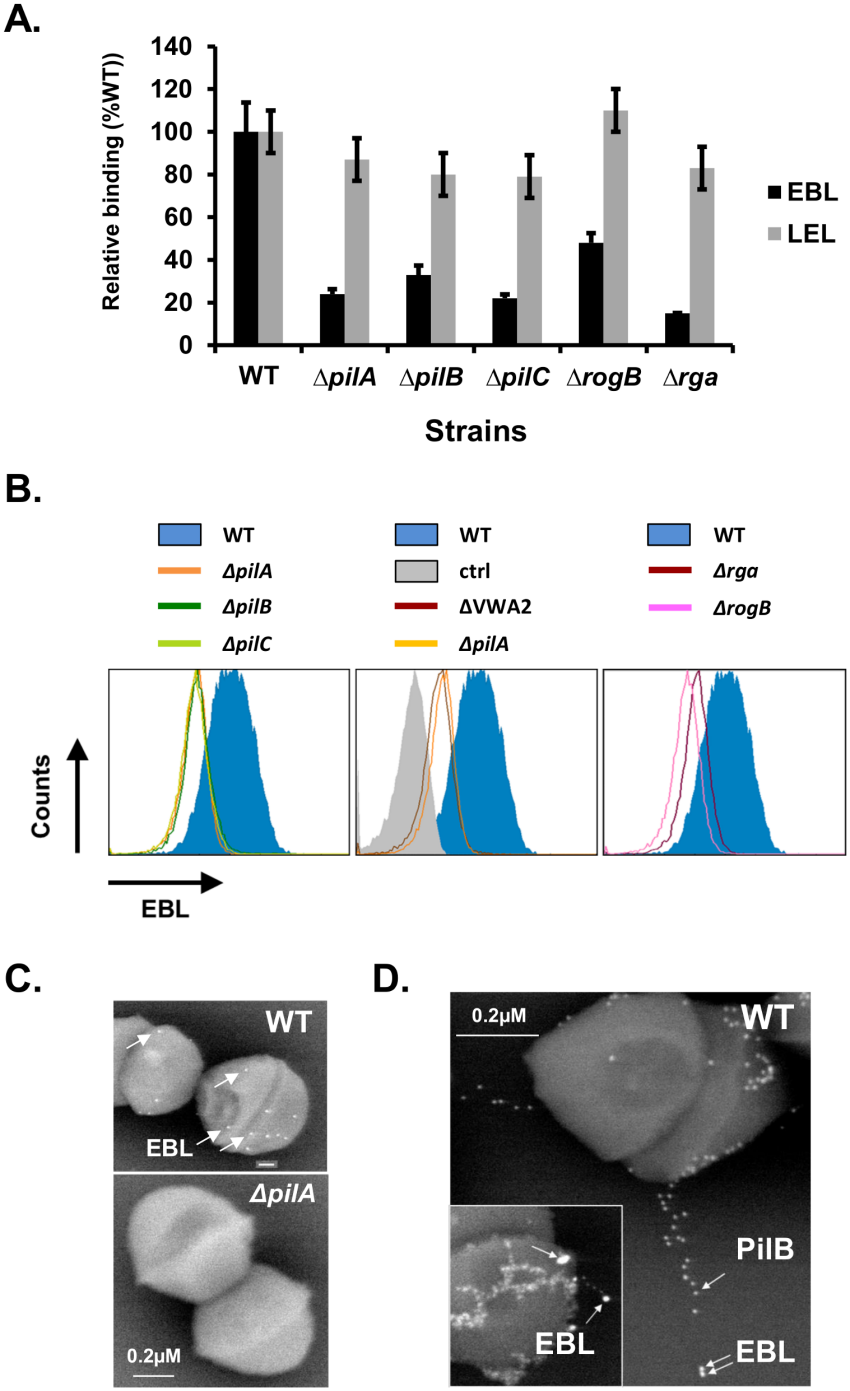
Pilus integrity is required for EBL binding. (A) Lectin binding assay of NEM316 wild-type (WT) and indicated isogenic in-frame deletion mutants immobilized on microtiter 96 wells plate incubated with biotinylated EBL or LEL (used as control). Values are mean ± SD normalized using signals measured with lectin (EBL) interacting with wild-type strain as reference. The assay was performed in duplicate and repeated at least two times independently. (B) Flow cytometry analysis of NEM316 wild-type (blue filled histogram) and isogenic in-frame deletion mutants (indicated colored line histogram) incubated with biotinylated EBL. (C) Immunoelectron microscopy (IEM) picture of the surface-bound biotinylated EBL on NEM316 wild-type (WT) and *ΔpilA* mutant. The bound lectin was detected using 10mm streptavidin conjugated gold particles. (D) Immunoelectron microscopy (IEM) picture of surface-bound biotinylated EBL and PilB pilus subunit on NEM316 wild-type (WT). The bound lectin was detected using 20mm streptavidin conjugated gold particles and PilB antibody conjugated to 10 nm gold particles. The major pilin PilB and EBL are marked by arrows.

To identify the pilus component(s) recognized by the EBL lectin, ELISA and flow- cytometry assays were carried out on isogenic *S*. *agalactiae* mutants: i) in-frame deletion mutants of *pilA*, *pilB*, and *pilC* encoding the tip adhesin, the major pilin, and the anchor pilin respectively [5, 7] and ii) mutants of *rga* and *rogB*, encoding major and minor regulatory genes [11] controlling transcription of PI-2a locus (Fig 2A and 2B). As shown in Fig 2A and 2B, binding of EBL lectin was significantly reduced in all five mutants when compared to the wild-type strain NEM316. The results suggested that any of the structural subunits constituting the PI-2a pilus could be glycosylated. PI-2A functionality is conferred by the PilA adhesin localized at the tip of the pilus and the integrity of the pilus fiber was shown to be important for optimal display of PilA on the bacterial surface [6]. Of note, PilA contains a central von Willebrand factor type A (VWA) domain exhibiting a conserved metal ion dependent adhesion site (MIDAS). To test the role of this domain in PilA, a new mutant with a much smaller deletion, named VWA2, was constructed in strain NEM316, in which only 9 amino acids including the critical aspartyl and seryl residues known to interact with divalent cations, were replaced by a 9 amino acids hemagglutinin epitope tag (HA tag), thereby allowing the detection of the mutant protein with specific anti-HA rat monoclonal antibody (data not shown). As shown for the previously characterized ΔVWA mutant (180 amino acids deletion; [6]), the VWA2 mutant also produced a very unstable PilA protein (see below). Consequently the ΔVWA2 mutant, like *ΔpilA*, was not recognized by the EBL lectin as shown in Fig 2B (middle panel).

Next, immunogold electron microscopy experiments were carried out to localize EBL binding on the pilus structure. Simple labeling with biotinylated EBL followed by 10nm gold-labeled streptavidin revealed surface binding on wild-type strain NEM316 and no binding on the isogenic *ΔpilA* mutant (Fig 2C). Double-labeling experiments were performed to detect simultaneously the pilus fiber using specific antibodies against PilB and EBL lectin and representative images are shown in Fig 2D. The inset displays a pilus fiber decorated with EBL lectin at its tip, a picture reminiscent of PilA localization previously described [5]. All together, these results pointed towards PilA as the pilus component recognized by the EBL lectin. Importantly, EBL binding was increased in the non-capsulated *ΔcpsE* and *Δneu* mutants (S2 Fig), an observation that correlates with the unmasking of PilA [6] in the absence of the capsular polysaccharide.

**Fig 3:**
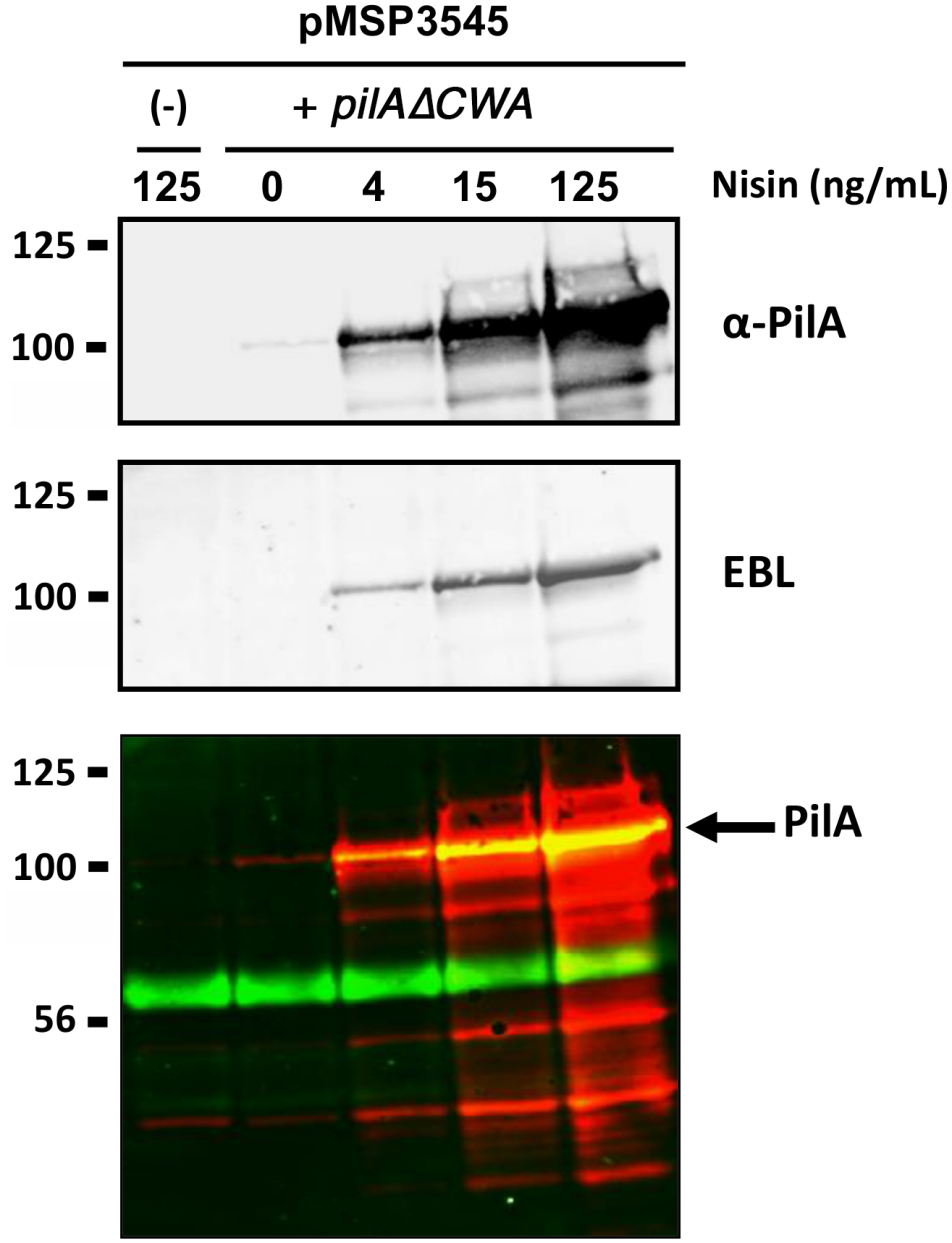
EBL binds to the tip adhesin PilA. A secreted form of PilA was produced using the nisin inducible expression system in NEM316 with vector pMSP3545-*pilA*(ΔCWA). Protein extracts of supernatant fraction from non-induced and nisin-induced overnight growth cultures at the indicated concentration and separated on 4%–15% Criterion XT SDS-PAGE and detected by blotting with specific anti-PilA polyclonal antibody (upper panel), biotinylated EBL (middle panel) or both (lower panel, where PilA and EBL labeling appears in red and green, respectively). The empty vector (-) induced at the highest nisin concentration tested was used as negative control. Equivalent amount corresponding to 500 μl of the initial culture was loaded in each well. The PilA subunit is indicated by a black arrow.

To further demonstrate that PilA produced in GBS was recognized specifically by EBL lectin, we overproduced a secreted form of PilA, PilAΔCWA (deletion of the C-terminal cell wall anchor sequence), in a dose-dependent manner using the nisin inducible expression system [15]. As shown in Fig 3, PilA production in the culture supernatants of NEM316 containing pMSP3545-*pilA*ΔCWA increased in a dose-dependent manner with nisin concentrations. No PilA-reactive band was detected in the control strain NEM316 harboring the empty vector pMSP3545. We used double fluorescence labeling to visualize simultaneously PilA (red signal) and EBL lectin (green signal) in order to demonstrate the direct binding of EBL to PilA (yellow signal). This experiment also revealed the presence of another secreted protein in GBS supernatant extracts of about 60 kDa recognized by the EBL lectin. Of note, the C-terminal histidyl-tagged PilA (6xHis Gbs1478) produced in *Escherichia coli* [5] did not bind EBL lectin (data not shown). These data suggest that glycosylation of PilA is specific to GBS and probably requires dedicated glycosyltransferases and/or oligosaccharyltransferases.

### Role of two putative N-glycosylated asparagine residues of PilA: mutant analysis

The lectin profiling shown in Fig 1B suggested that PI-2a pilus is most likely modified with *N-*linked sugars. Different mass spectrometry methods [10] have been tested to identify the sugar-modified residues on either whole PI-2a pili or streptococcal overexpressed PilA preparations. All our attempts have failed so far and several reasons could be proposed: i) PI-2a pili preparations are heterogeneous for glycan composition and highly resistant to proteases; ii) only a small fraction of the overproduced PilA is modified with sugars; furthermore, the glycosylation profile of monomeric PilA overexpressed in GBS supernatant, appears different from that identified in polymerized pili (data not shown).

**Fig 4:**
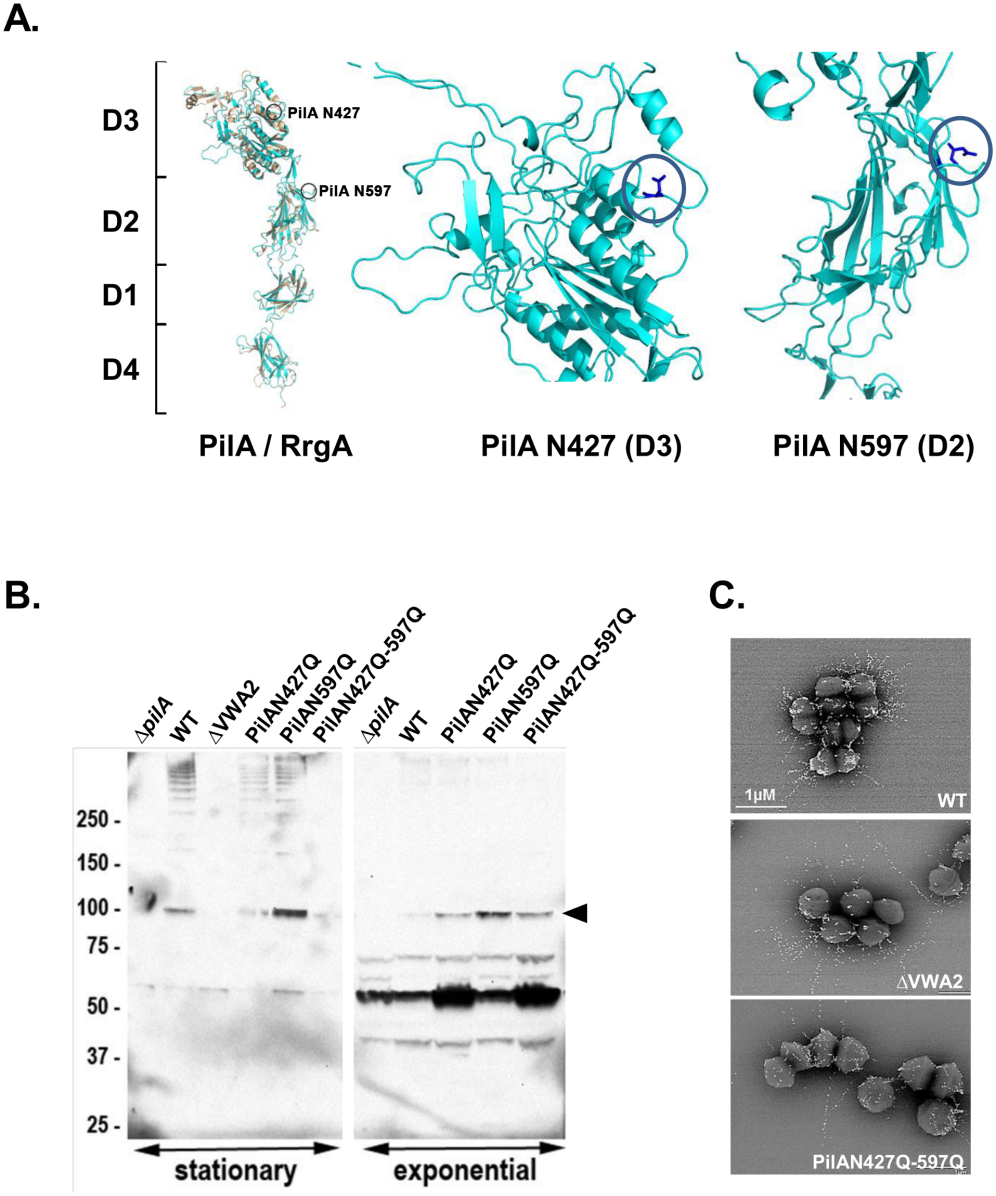
Role of putative *N*-glycosylated asparagyl residues in PilA stability. (A) Structural modeling of PilA (blue ribbon) compared to the *S*. *pneumoniae* homolog RrgA (orange ribbon). Domain organization (D1 to D4) appears to be conserved, position of *in silico* putative predicted *N*-glycosylated aspargyl residues N427 and N597 in domains D3 and D2 are indicated (blue circle). (B) Proteins anchored to the cell-wall were isolated from *S*. *agalactiae* strain NEM316 and its isogenic *pilA* mutants harvested at both exponential and stationary growth phase, separated on 4%–12% Criterion XT SDS-PAGE gel, and detected by immunoblotting with specific anti-PilA antiserum. Equivalent amount corresponding to 500 μl of the initial culture was loaded in each well. The PilA monomer is indicated by a black arrow. The high-molecular-weight species correspond to PilA polymers while the lower band at 50 kDa is most likely a degradation product. (C) Immuno-electron-microscopy (IEM) analyses of the pilus subunits PilB. *S*. *agalactiae* wild-type strain NEM316 (WT) and its isogenic pilus mutants (ΔVWA2, and PilAN427Q-N597Q) were incubated with rabbit polyclonal antibody raised against PilB. Antibodies were conjugated to 10 nm gold particles.

*In silico* analysis of the PilA 901 amino-acid long sequence for post-translational modifications (NetNGlyc 1.0 prediction tool) revealed three predicted *N-*glycosylated asparagine located at positions 107, 427, and 597. By comparison with the tridimensional structure of the related pilus associated adhesin RrgA from *S*. *pneumoniae* [16], two asparagyl residues were found conserved and exposed in PilA (Fig 4A and S4 Fig). We thus replaced each of these asparagyl residues of PilA into a non-modifiable glutamyl residue (N427Q, N597Q), and then combined both mutations (N427Q-N597Q) on the chromosome of strain NEM316. Western blot analysis of PilA expression in exponentially growing bacteria revealed the presence of monomeric PilA (≈100 kDa) in WT, PilAN427Q, PilAN597Q and PilAN427Q-N597Q, but not in the *ΔpilA* strain used as control (Fig 4B, right part). Non-specific bands at lower molecular weight (below 75 kDa) also detected in the *ΔpilA* mutant served as internal loading controls. In contrast, typical high molecular weight species containing PilA were detected in the cell-wall extracts of WT, PilAN427Q, and PilAN597Q grown to stationary cultures. However, only a very faint band corresponding to monomeric PilA and no higher polymers were detected in the double mutant PilAN427Q-N597Q. Of note, no PilA reactive species could be detected in the cell wall extracts of the ΔVWA2 mutant demonstrating the key role of metal binding for PilA stability. We also examined PilA levels in membrane extracts of exponentially growing bacteria (Fig A in S5 Fig). Similar amounts of PilA were detected in NEM316 WT, PilAN427Q, PilAN597Q, PilAN427Q-N597Q, but not in the *ΔpilA* mutant. Interestingly, PilA destabilization was revealed in the ΔVWA2 mutant through the appearance of a PilA-specific band at approximately 50 kDa (Fig A in S5 Fig). Flow-cytometry analyses of various PilA mutants further confirmed similar levels of PilA expression in the single and double asparagyl mutants as compared to the WT strain. As expected, PilA was highly unstable in the ΔVWA2 mutant (Fig B in S5 Fig).

We previously demonstrated that, in the absence of PilA, very long pili polymers were formed [5]. We thus visualized PilB containing fibers by immunogold electron microscopy in the WT, VWA2 and PilAN427Q-N595Q (Fig 4C). As expected from our previous study [6], significantly longer pili were found in both ΔVWA2 and PilAN427Q-N595Q (mean length 700–950 nm) as compared to the WT (250–350 nm) further pointing to PilA destabilization in these mutants. Collectively, our results suggests that glycosylation of PilA may be important for protein stability and may confer some functional properties to the pilus.

**Fig 5:**
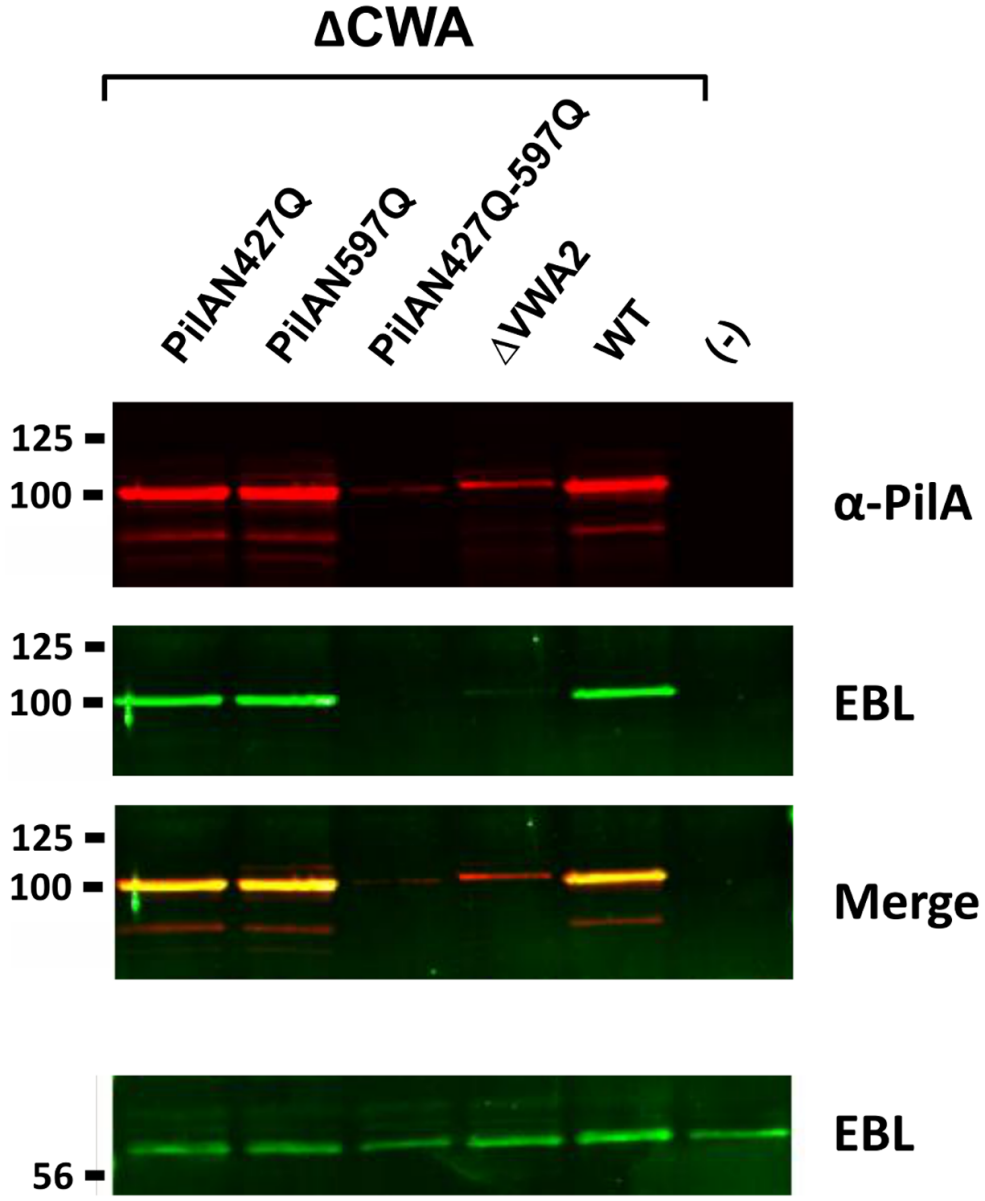
Role of putative *N*-glycosylation asparagyl residues in pilus integrity. Secreted form of both PilA (WT) and mutated forms in putative *N*-glycosylated sites (N427Q, N597Q, N427Q-N597Q) were produced using the nisin inducible expression system in NEM316. Protein extracts of supernatant fraction from nisin-induced (15 ng/mL) late exponential growth phase cultures were separated on 4%–15% Criterion XT SDS-PAGE and detected by blotting with specific rabbit anti PilA antiserum (red), biotinylated EBL (green) or both (merge). Equivalent amount corresponding to 500 μl of the initial culture was loaded in each well. Empty vector (-) induced at the same nisin concentration was used as negative control. A vector producing a previously described non-stable mutated form of PilA (ΔVWA2) was also included as control. The signal obtained with the ~60kDa secreted protein that is able to efficiently bind EBL has been included is shown as loading control.

Finally, overexpression of the various PilA mutant forms was performed using the nisin- inducible vector pMSP3545. Western blot analysis of concentrated supernatant extracts is shown in Fig 5A. A strong PilA reactive band was detected in the WT, N427Q and N597Q single mutants. In contrast, only a faint band was detected in the double N427Q-N497Q and ΔVWA2 mutants. Recognition by EBL was also tested revealing that both simple mutants N427Q and N597Q were recognized. No EBL signal was detected for the double mutant N427Q-N597Q. However, this result is not conclusive and may simply reflect the fact that PilA protein was strongly destabilized in this mutant. Thus, a definitive proof of the *N*-glycosylation of the PilA subunit will require further investigations.

## Discussion

This work was part of a large effort to identify surface glycoproteins of *S*. *agalactiae* using our reference strain NEM316 [17]. Our laboratory previously characterized the PI-2a pilus locus [5, 6] and the accessory SecA2 locus encoding the Srr1 glycoprotein [8]. We serendipitously found that the transcription of PI-2a locus was strongly activated by Rga encoded by *gb1530*, located immediately upstream of *srr1* (*gbs1529*) gene [11]. This unsuspected relationship led us to question the glycosylation status of pili. In support of this hypothesis, PilE the major pilin of *N*. *gonorrhoeae* type IV pili, was shown to be glycosylated by mass-spectrometry [18].

Glycan profiling and analysis of monosaccharide compositions of native affinity-purified PI-2A pilus polymers from cell wall extracts of GBS strain NEM316 indicated that these surface appendages are modified with carbohydrates (Fig 1, Table 1). We next provided indirect evidences suggesting that it is probably PilA, the pilus associated adhesin, which is glycosylated to display *N*-acetylneuraminic (also known as sialic acid, Neu5Ac) attached to terminal galactose by a **α**-2, 6 linkage, most likely on an asparagyl residue (Figs 2–5). Another secreted protein of about 60 kDa in strain NEM316 was also recognized by EBL (Fig 3). This protein may correspond to the abundantly secreted Bsp protein [19] or eventually to PcsB protein whose counterpart in *S*. *mutans*, IDG-60, has been proposed to be glycosylated [20].

Interestingly, terminal sialic acid linked to galactose by a **α**-2, 3 linkage is characteristic of nearly all GBS capsular polysaccharides and was shown to inhibit the activation of the alternative complement pathway [21]. This conserved disaccharide constitutes a motif which is very similar to that displayed by sialyl Lewis antigen and related carbohydrate epitopes found in human proteins. It is hypothesized that this molecular mimicry is used by GBS to avoid recognition by the host immune system. Recently, it has been shown that GBS interacts with an inhibitory Siglec-5 (sialic acid- binding immunoglobulin-like lectin) receptor through sialic acid mimicry to blunt innate and immune responses *in vivo* [22]. Sialylated GBS was also shown to interact with sialoadhesin, a macrophage receptor, which is critical for GBS phagocytosis and clearance [22].

All our attempts to identify by mass-spectrometry techniques the amino acid residues modified in PilA and the nature of sugar attached to it have failed so far. First, we noticed that when overexpressing PilA in GBS, its glycan profile and composition was slightly different from that obtained with the whole pili polymers, suggesting that glycosylation process is tightly coupled to secretion/polymerization machineries, and may require a proper expression ratio between sugar donor and acceptor (i.e. glycosyltransferase and PilA). Second, when comparing SEM micrographs of GBS NEM316 labeled with either PilB/PilA or PilB/EBL, we found that the proportion of EBL clearly localized at the tip (3 over 100 PilB pili counted) was low compared to the number of PilA detected at the tip of PilB pilus (25 over 100 PilB pili counted), suggesting that only a small proportion of PilA is recognized by the EBL lectin. Heterogeneity of glycosylation sites may also be tremendous as exemplified with Srr1 [10]. This glycan heterogeneity probably represents the biggest challenge and technical limit in the analysis of bacterial glycoproteins.

Of note, PilA deleted of its cell wall anchor domain and overexpressed in *Lactococcus lactis* strain NZ9000 was found in the supernatant and recognized by EBL lectin (data not shown). Interestingly, another secreted protein from *L*. *lactis* was recognized by the EBL and, based on its apparent molecular mass, was tentatively identified as Usp45 glycoprotein [23]. This result indicates that the glycosyltransferases mediating PilA glycosylation are also present in *L*. *lactis*. Indeed, genome analysis for putative glycosyltransferases in GBS and lactococci revealed a similar repertoire of about 30 conserved genes that were further analyzed using the CAZY database (http://www.cazy.org/).

As indicated in the results section, recombinant histidyl-tagged PilA produced and purified from the cytoplasmic compartment in *E*. *coli* BL21 (DE3) was not glycosylated (data not shown) suggesting the existence of pilus-specific glycosytransferases and/or oligosaccharyltransferases which are absent in the *E*. *coli* genome. However, we cannot rule out the possibility that PilA was not glycosylated because it was not secreted. It is worth noting that a large cluster of glycosyltransferases is present (*gbs1480-1494*) in the vicinity of the PI-2a chromosomal locus (gbs1479-1474). This genetic locus was proposed to encode the group B carbohydrate (GBC) antigen [24].

The only sialyltransferase characterized to date in GBS is encoded by *cpsK* (Chaffin et al., 2005). CpsK is required for the sialylation of capsular polysaccharide but also for optimal capsule polymerization and expression (Chaffin et al., 2005). It would be tempting to speculate that glycosyltransferases participating in the synthesis of major polysaccharides structures (capsule and GBC) extend their activity to the glycosylation of surface proteins.

Our data suggest that abundant surface polymers such as the PI-2a pili in GBS strain NEM316 are sialylated and thus protected from the host immune recognition. It remains to be shown whether the sialylation of PilA can also modulate the host immune responses.

## Materials and Methods

### Bacterial strains, media, and growth conditions

*S*. *agalactiae* strain NEM316 belongs to the capsular serotype III (ST-23) and was entirely sequenced [17]. *Escherichia coli* XL1-blue competent cells (Agilent) were used for cloning experiments. *S*. *agalactiae* was cultured in Todd-Hewitt (TH) broth or agar (Difco Laboratories, Detroit, MI) and *E*. *coli* in Luria-Bertani (LB) medium. Unless otherwise specified, antibiotics were used at the following concentrations: for *E*. *coli*—erythromycin, 150 μg/ml; for *S*. *agalactiae*—erythromycin, 10 μg/ml. *S*. *agalactiae* liquid cultures were grown at 37°C in standing filled flasks. Strains and plasmids used in this study are detailed in S2 Table.

### General DNA techniques

Standard recombinant techniques were used for nucleic acid cloning and restriction analysis [25]. Plasmid DNA from *E*. *coli* was prepared by rapid alkaline lysis using the Qiaprep Spin Miniprep kit (Qiagen). Genomic DNA from *S*. *agalactiae* was prepared using the DNeasy Blood & Tissue kit (Qiagen). PCR was carried out with Phusion High-Fidelity DNA polymerase following the manufacturer’s recommendations (Thermo Scientific). Amplification products were purified on Qiaprep PCR purification kit (Qiagen) and sequenced with an ABI 310 automated DNA sequencer, using the ABI PRISM dye terminator cycle sequencing kit (Applied Biosystems).

### Construction of mutants

Replacement of amino acids Aspargine 427 and/or 597 to glutamine in *pilA* (*gbs1478*) were carried out by overlap extension PCR using the primers containing the desired mutations pilAN427QF–pilAN427QR and pilAN597QF–pilAN597QR) as described previously [5]. The sequences (5′ to 3′) of the primers used to introduce the mutations in NEM316 chromosome were: pilAN427QF_Eco, AAATAGAATTCCCTTCAAACTAAGTTCACAATTCAG; pilAN427QF, GGCTACCTGCAGAAGTCCAACTTTCTACTTACTGATAAGCCCGAG; pilAN427QR: GTTGGACTTCTGCAGGTAGCCATTTTTTTTCATTTGTTCAAATTGGC; pilAN427QF_Bam, GTTATGGATCCATCTGAAAGTTCAAAAGC; pilAN597Q_Eco: TAATTgaattCTTACTGATAAGCCCGAGGAT pilAN597QF: AAGGAGCACTCCATCGTCCAGGGAACTATAGAAGATCCTATG; pilAN597QR: CTGGACGATGGAGTGCTCCTTTGTTAAAACCTTTTCAAATTG, and pilAN597Q_Pst TGAAGctgcagCGTAGCTCCTTTGAGAAGCA. The same strategy was used to construct the NEM316: PilAVWA2 mutant in which 9 amino acids (including the critical aspartate and serine residues known to interact with divalent cations) of the Von Willebrand Adhesion domain were replaced by a 9 amino acids hemagglutinin epitope tag. Primers O1-O2 and O3- O4 were used. The sequences (5’ to 3’) are as follows: O1, CCCAAGAATTCGCTGAGCTAACAGGTGAAGCTA; O2, GGCGTAGTCGGGGACGTCGTAGGGGTACTTTTGTTTGTCCACTGGTTTTAC; O3, TACCCCTACGACGTCCCCGACTACGCCTCTAACTCAATGAATAACGATGGC; O4, ATAGCGGATCCTCTTGTTGGAACACCATCAG.

To carry out chromosomal codon replacements, appropriate PCR fragments were cloned into the thermosensitive shuttle plasmid pG1 (S2 Table). Electroporation of GBS strains and allelic exchange were performed as described previously [5]. Mutations were checked by PCR and confirmed by sequencing on the chromosome.

### Pili purification

*S*. *agalactiae pili* NEM316 were purified from mutanolysin treated cell-wall extract by affinity chromatography. Briefly, cell-wall extracts from a large culture (2 L) of strain NEM316 grown overnight at 37°C were prepared as previously described [5] and loaded on a 5 mL Hi-Trap NHS activated HP column formerly conjugated with specific anti-PilB (Gbs1477) polyclonal antibody following manufacturer instructions (GE healthcare). Approximately 20 mL of the lysate was applied slowly with a peristaltic pump and passaged several times on the column for 8 h at 4°C. The flow-through was collected for analysis. After several washes at high salt concentration (500 mM NaCl), elution was performed using 100 mM glycine/HCl pH 2.5. Acidic pH of eluted fractions was immediately neutralized using Tris 1 M pH 8.8. Presence and purity of *pili* in collected fractions was analyzed by SDS-PAGE using criterion-XT 3–9% gel (Biorad) and silver staining. Fractions containing highly purified pili were pooled and used for glycan profiling. Absence of contamination of the pili fraction with type III capsular polysaccharide was checked by dot-blot analysis using specific polyclonal antibodies (S1 Fig).

### Glycan profiling

The lectins were directly printed in triplicates on Biomat (Rovereto, Italy) fluorescent black 96 wells plates according to GLYcoDIAG technology (GlycoDIAG, Orleans, France). Each lectin was immobilized on high binding capacity microtiter wells at 10–25μg/mL (depending on the lectin) and several wells without lectin were added as negative controls. Quantity and contact duration were optimized for each lectin. The specificity of the lectins used in this study is listed in S1 Table. After washing and saturation with bovine serum albumin (BSA), plates were used immediately or freeze-dried. Each produced batch was controlled by using a set of reference glycoproteins and neoglycoproteins (BSA linked to mono or disaccharides) to ensure the repeatability of the assay itself. Purified PI-2A pili were first biotinylated and applied to the lectin wells at (20 μg/mL). Then HRP streptavidin was added and the enzymatic activity was revealed by colorimetric assay (OD450 nm). Washing and/or saturation of the wells were performed between each of these steps as previously described [26]. Each value was calculated by removing the background level (control wells without lectin). The results were normalized to the binding of EBL (arbitrarily fixed to 100%).

### HPAEC analysis

Dosage of monosaccharides and sialic acids were performed after acid hydrolysis of affinity purified pili treated with either 100 mM HCl for one hour at 80°C for sialic acids dosage or 2 M TFA for 4 h at 100°C for monosaccacharides dosage. Hydrolysate products were dried using vacuum concentrator and resuspended in water before ion chromatography analysis using CarboPac PA1 column coupled with pulsed amperometric detection (Dionex). Fifty and 25 μg of hydrolysate were loaded in the column for quantification of monosaccharides and sialic acids, respectively.

#### Production of secreted form of PilA, preparation of supernatant protein extracts and blotting analysis

Nisin-inducible expression of wild-type and mutated secreted forms of PilA that do not contain the C-terminal cell wall anchor region (Δcwa) was obtained by cloning each mutated form of *pilA* (gbs1478) into pMSP3545 vector [15, 27]. Each gene was PCR amplified from genomic DNA of wild-type and *pilA* mutants using primers pilA-pMSP-F and pilA-pMSP-R. The sequences (5′ to 3′) of the primers are: pilA-pMSP-F GCGCGCCATGGGAAAATACCAAAAATTTTCTAAAATATTGACG and pilA-pMSP-R GCGCGTCTAGAAATTCCTTTTGGTGGAATATGCGTGTTGG. Purified PCR products were digested with *Nco*I-*Xba*I and ligated into pMSP3545 cut with the same enzymes. The five different plasmids with PilA WT, N427Q, N597Q, N427/597Q or VWA2 were then introduced in NEM316 strain by electroporation. Supernatant proteins containing the recombinant secreted PilA were prepared as follows: bacterial cultures were grown overnight at 37°C in TH medium supplemented with 10μg/mL of erythromycin and nisin (Sigma-Aldrich) at the indicated concentrations (0–125 ng/mL). Supernatants were harvested in late exponential phase cultures and protein extracts were prepared as previously described ([28]. Secreted PilA were analyzed by Western immunoblotting as follows. Proteins were boiled in Laemmli sample buffer, separated by SDS-PAGE on 4–12% Tris-Acetate Criterion XT gradient gels and transferred to nitrocellulose membrane (Hybond-FL, Amersham). PilA was detected using specific polyclonal antibodies previously described and Dylight 680-conjugated anti-rabbit secondary antibodies (Zymed). EBL binding experiments on PilA bound to the membrane were performed using biotinylated-EBL lectin and Dylight 800-conjugated streptavidin (Zymed). Blocking was a critical step and results shown were obtained using the SuperBlock^®^ Blocking Buffer in PBS (Thermo Scientific). Image capture and analysis were done on Odyssey NIR fluorescence imaging system (LICOR).

#### Immunogold electron microscopy

For scanning electron microscopy (SEM), bacteria were collected after overnight growth, fixed, and stained either with rabbit anti-PilA, IgG followed by anti-rabbit secondary antibody conjugated to 10 nm colloidal gold or with rabbit anti-PilB and biotynilated EBL lectin followed by anti-rabbit secondary antibody conjugated to 10 nm colloidal gold and streptavidin conjugated to 20 nm colloidal gold as previously described [6].

### Fluorescence-activated cell sorter analysis (FACS)

To analyze EBL binding on various GBS strains by flow-cytometry, bacteria grown overnight in TH at 37°C, were collected and washed twice in phosphate buffered saline (PBS) and incubated for 15–30 min with biotinylated EBL lectin (Vector Laboratories) at 10 μg/mL in PBS-BSA 1%. After three washings with PBS, samples were incubated for 30 min with AlexaFluor488-conjugated streptavidin (Molecular Probes, Invitrogen). Cells were washed and resuspended in PBS. Samples were fixed with PBS-PFA 1% for 10 min, washed with PBS and acquired by a Beckman Coulter Cytomics FC500 apparatus and data were analyzed using Cytomics RXP software.

## Supporting Information

S1 Fig Purified pili fractions are free of capsular polysaccharide contamination. (A) To demonstrate the absence of contamination of the *pili* fractions by type III capsular polysaccharides, dot-blot analysis were performed on mutanolysin cell-wall extracts (CW) and purified PI-2a fraction using specific polyclonal antibodies raised against type III capsular polysaccharides (α-CapIII). Detection of *pilus* subunits PilA, B and C in the purified PI-2a fraction by specific polyclonal antibodies was included as control. (B) Specificity of antibodies raised against type III capsular polysaccharide was verified by performing dot-blot analysis on whole bacteria using NEM316 wild-type strain (WT), the non-capsulated mutant *ΔcpsE* deleted in gene responsible for the synthesis of the polysaccharide repeating units, the *ΔneuBCDA* mutant deleted in genes responsible for the synthesis of sialic acid present in GBS capsular polysaccharides and the *ΔpilA* mutant. Result obtained using specific anti-PilA antibodies on the same whole bacteria was included as control.
(TIFF)

S2 Fig EBL binds better non-capsulated GBS mutant. Flow cytometry analysis of NEM316 wild-type (WT) and isogenic in-frame deletion mutants *ΔcpsE* and *ΔneuBCDA* (indicated colored line histogram) incubated with biotinylated EBL (left panel) and specific anti-PilA antiserum (right panel). EBL binds better to non-capsulated mutants probably due to the unsmaking of streptococcal adhesins (e.g. PilA) binding this lectin.
(TIFF)

S3 Fig Immobilized purified *pili* from *S*. *agalactiae* NEM316 binds EBL specifically. PI-2a native pili were loaded on 96 wells plate at 10 and 50 ng per well and tested for recognition by 10 different lectins displaying various specificities (see S2 Table). As unrelated control, we used the Bovine Serum Albumin loaded at 100 ng per well. A dose-dependent and specific signal was detected with the Elderberry Bark Lectin (EBL), also known as Sambuca Nigra (SNA) lectin, which binds preferentially to sialic acid attached to terminal galactose in **α** -2, 6 and to a lesser extent in **α**-2, 3 linkage (Fig 1B).
(TIFF)

S4 Fig Sequence alignment of GBS NEM316 PilA and RrgA of *S*. *pneumoniae* TIGR4. PilA sequence (Genbank accession number WP_001233990.1) from GBS NEM316 and RrgA (Genbank accession number AAK74622.1) from *S*. *pneumoniae* TIGR4 were aligned using ClustalW2 server (http://www.ebi.ac.uk/Tools/msa/clustalw2/). An (*) indicates positions which have a single, fully conserved residue. A (:)indicates conservation between groups of strongly similar properties. A (.) indicates conservation between groups of weakly similar properties. Amino acids corresponding to RrgA D1 to D4 defined domains are colored as follow: D1 (green), D2 (blue), D3 (pink) and D4 (orange). Potential N-glycosylated residues (PilAN427 and 597, in red) are shown in box. Residues corresponding to LPXTG-anchoring signal are underlined.
(TIFF)

S5 Fig PilA levels in membrane extracts of exponentially growing bacteria. (A) Proteins from membrane were isolated from *S*. *agalactiae* strain NEM316 and its isogenic *pilA* mutants harvested during exponential growth phase, separated on 4%–12% Criterion XT SDS-PAGE gel, and detected by immunoblotting with specific anti PilA antiserum. (B) Flow cytometry analysis of NEM316 wild-type (WT) and isogenic in-frame deletion mutants (indicated colored line histogram) incubated with specific anti PilA antiserum.
(TIFF)

S1 Table Specificity of lectins used in this study. (DOCX)

S2 Table Bacterial strains and plasmids used in this study. (DOCX)
