# Supplementary figures and images for: Evidence for the Sialylation of PilA, the PI-2a Pilus-Associated Adhesin of Streptococcus agalactiae Strain NEM316
Source: PLoS One. 2015 Sep 25;10(9):e0138103. doi: 10.1371/journal.pone.0138103 (PMC4583379; doi:10.1371/journal.pone.0138103)

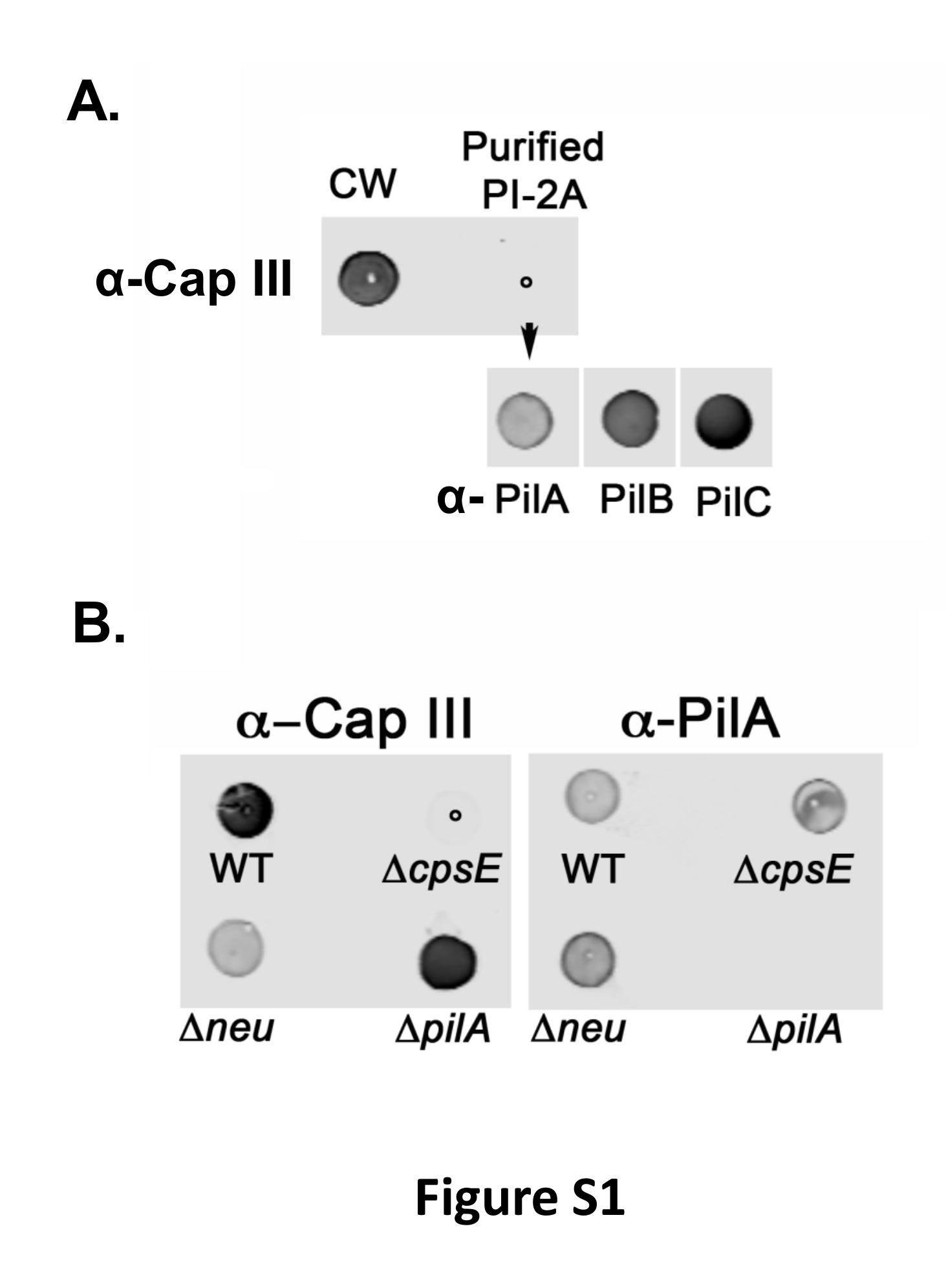

Supplement: S1 Fig — (A) To demonstrate the absence of contamination of the pili fractions by type III capsular polysaccharides, dot-blot analysis were performed on mutanolysin cell-wall extracts (CW) and purified PI-2a fraction using specific polyclonal antibodies raised against type III capsular polysaccharides (α-CapIII). Detection of pilus subunits PilA, B and C in the purified PI-2a fraction by specific polyclonal antibodies was included as control. (B) Specificity of antibodies raised against type III capsular polysaccharide was verified by performing dot-blot analysis on whole bacteria using NEM316 wild-type strain (WT), the non-capsulated mutant ΔcpsE deleted in gene responsible for the synthesis of the polysaccharide repeating units, the ΔneuBCDA mutant deleted in genes responsible for the synthesis of sialic acid present in GBS capsular polysaccharides and the ΔpilA mutant. Result obtained using specific anti-PilA antibodies on the same whole bacteria was included as control. (TIFF) [file pone.0138103.s001.tiff]

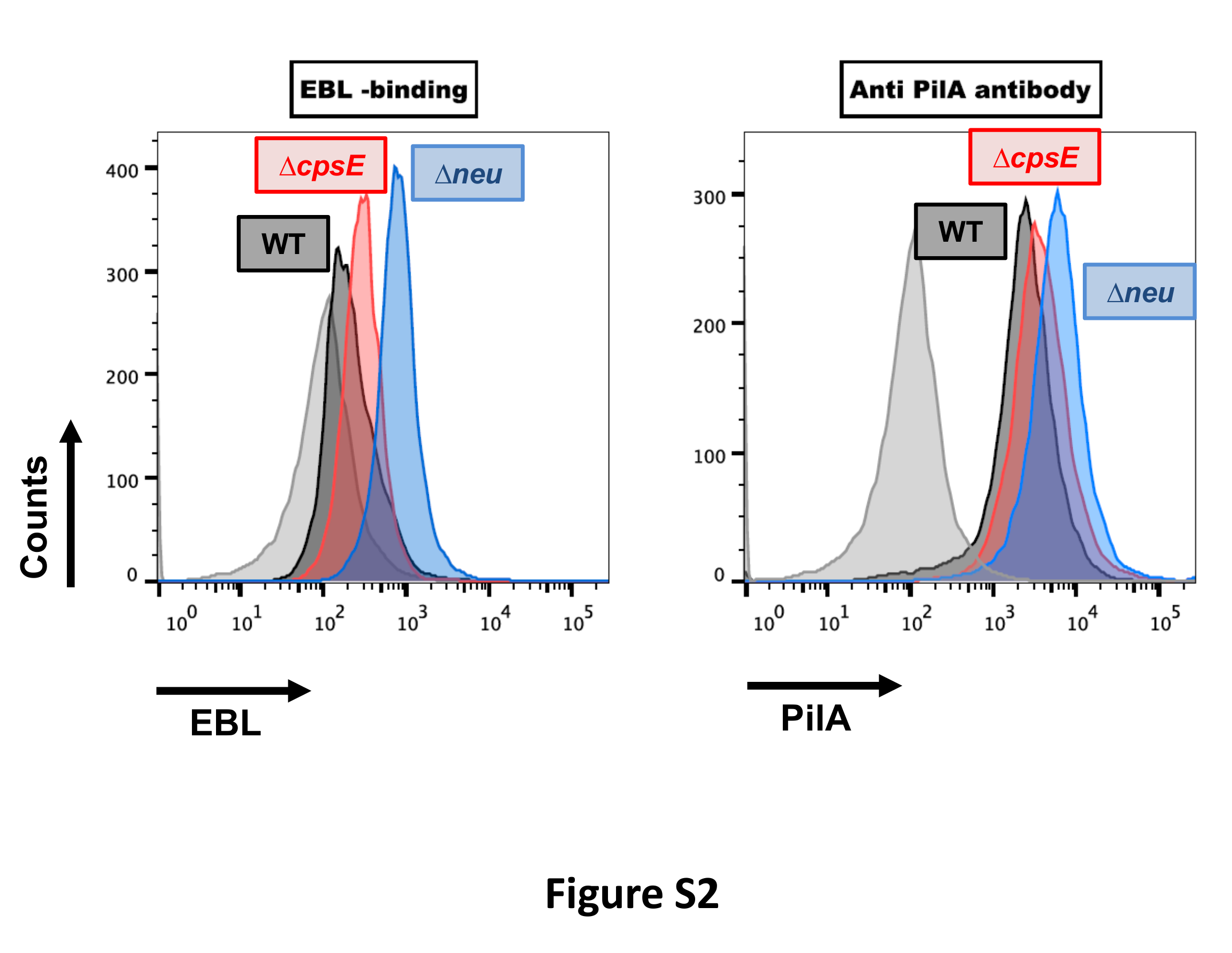

Supplement: S2 Fig — Flow cytometry analysis of NEM316 wild-type (WT) and isogenic in-frame deletion mutants ΔcpsE and ΔneuBCDA (indicated colored line histogram) incubated with biotinylated EBL (left panel) and specific anti-PilA antiserum (right panel). EBL binds better to non-capsulated mutants probably due to the unsmaking of streptococcal adhesins (e.g. PilA) binding this lectin. (TIFF) [file pone.0138103.s002.tiff]

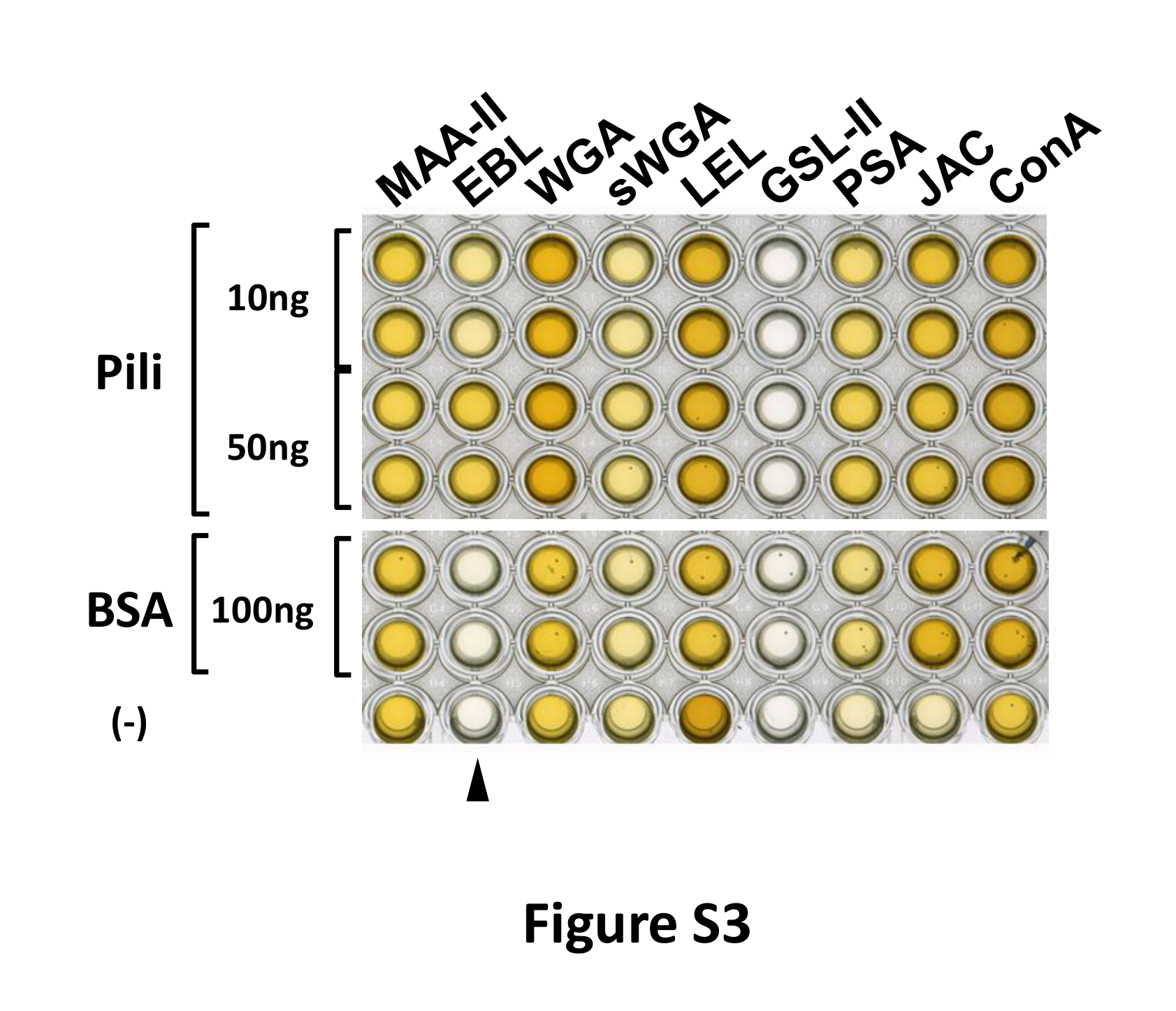

Supplement: S3 Fig — PI-2a native pili were loaded on 96 wells plate at 10 and 50 ng per well and tested for recognition by 10 different lectins displaying various specificities (see S2 Table). As unrelated control, we used the Bovine Serum Albumin loaded at 100 ng per well. A dose-dependent and specific signal was detected with the Elderberry Bark Lectin (EBL), also known as Sambuca Nigra (SNA) lectin, which binds preferentially to sialic acid attached to terminal galactose in α -2, 6 and to a lesser extent in α-2, 3 linkage (Fig 1B). (TIFF) [file pone.0138103.s003.tiff]

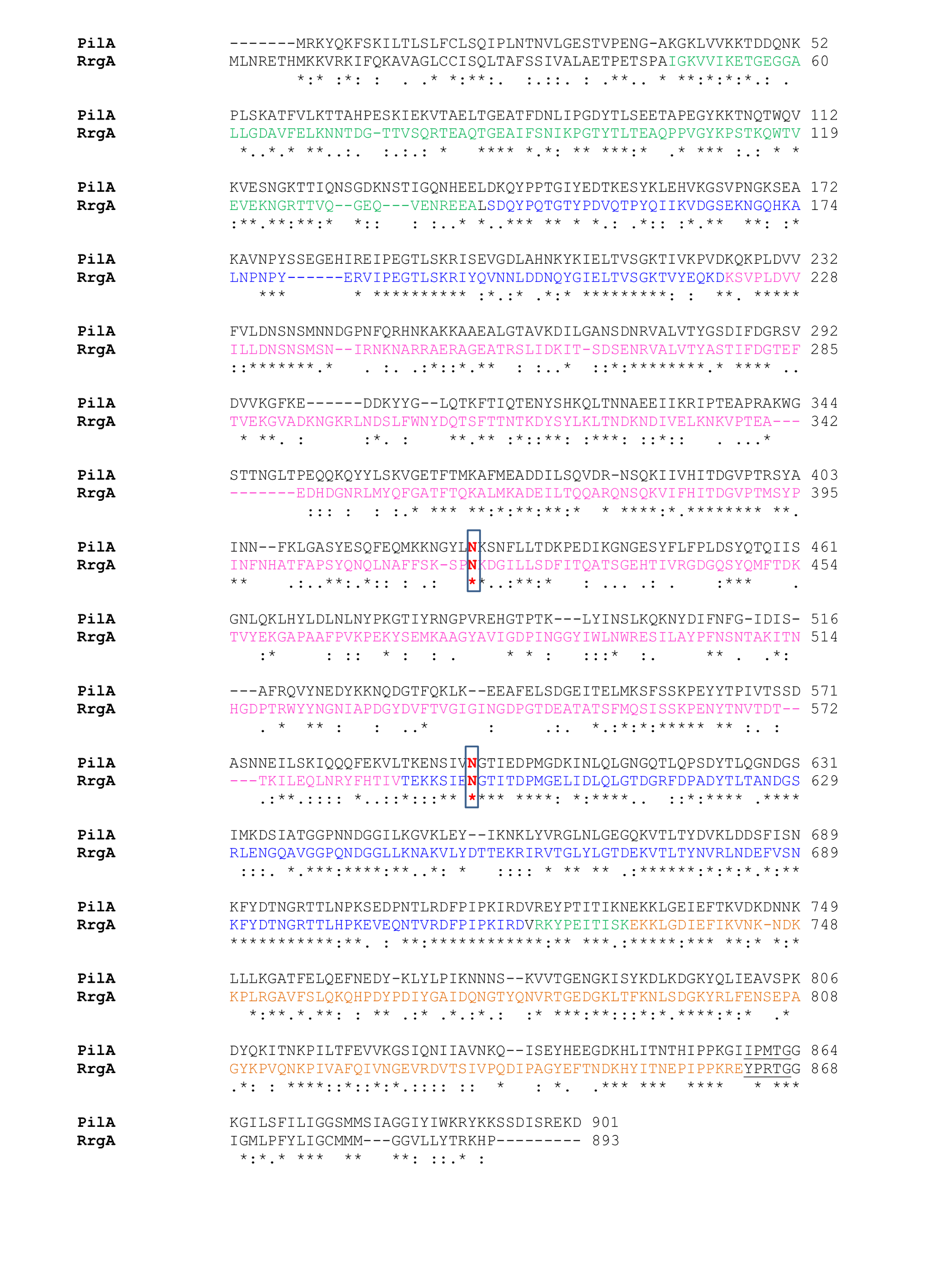

Supplement: S4 Fig — PilA sequence (Genbank accession number WP_001233990.1) from GBS NEM316 and RrgA (Genbank accession number AAK74622.1) from S. pneumoniae TIGR4 were aligned using ClustalW2 server (http://www.ebi.ac.uk/Tools/msa/clustalw2/). An (*) indicates positions which have a single, fully conserved residue. A (:)indicates conservation between groups of strongly similar properties. A (.) indicates conservation between groups of weakly similar properties. Amino acids corresponding to RrgA D1 to D4 defined domains are colored as follow: D1 (green), D2 (blue), D3 (pink) and D4 (orange). Potential N-glycosylated residues (PilAN427 and 597, in red) are shown in box. Residues corresponding to LPXTG-anchoring signal are underlined. (TIFF) [file pone.0138103.s004.tiff]

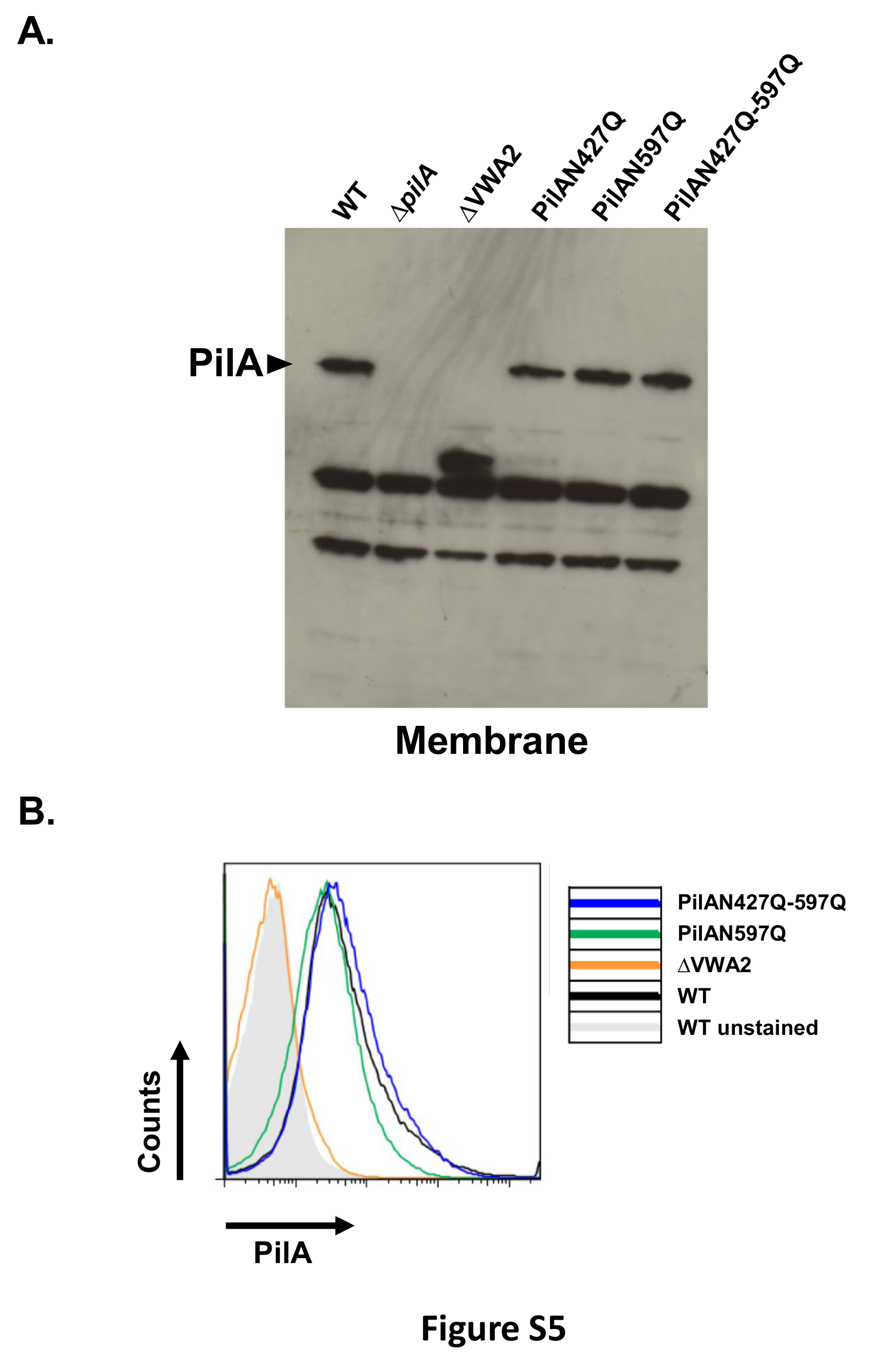

Supplement: S5 Fig — (A) Proteins from membrane were isolated from S. agalactiae strain NEM316 and its isogenic pilA mutants harvested during exponential growth phase, separated on 4%–12% Criterion XT SDS-PAGE gel, and detected by immunoblotting with specific anti PilA antiserum. (B) Flow cytometry analysis of NEM316 wild-type (WT) and isogenic in-frame deletion mutants (indicated colored line histogram) incubated with specific anti PilA antiserum. (TIFF) [file pone.0138103.s005.tiff]
