# Supplementary material for: Evidence for the Sialylation of PilA, the PI-2a Pilus-Associated Adhesin of Streptococcus agalactiae Strain NEM316
Source: PLoS One. 2015 Sep 25;10(9):e0138103. doi: 10.1371/journal.pone.0138103 (PMC4583379; doi:10.1371/journal.pone.0138103)
Supplement: S1 Table — (DOCX) [file pone.0138103.s006.docx]

**S1 Table.** Specificity of lectins used in this study^1^

| **Lectins** | **Abbreviation** | **Inhibitor monosaccharide** | **Reported Glycan structure specificity** |
| --- | --- | --- | --- |
| *Pisum sativum* | PSA | Mannose, Glucose | α-linked mannose-containing oligosaccharides, with an *N*  acetylchitobiose-linked α-fucose- |
| *Galanthus nivalis* | GNA | Mannose | structures containing (α-1,3) mannose residues, High mannose type N-glycans (bind manopentaose), terminal mannoses  no binding to α-linked glucose. |
| *Bauhinia purpurea* | BPA | *N*-acetylgalactosamine | galactosyl (β-1,3) *N*-acetylgalactosamine structures (T-antigen),  oligosaccharides with a terminal α-linked *N*-acetylgalactosamine can also bind |
| *Helix pomatia* | HPA |  | terminal N-acetyl-α-D-galactosamine residues. |
| *Sophora japonica* | SJA |  | carbohydrate structures terminating in *N*-acetylgalactosamine and galactose residues. GalNacβ3 or 4, GalNAcβ6Gal is a better inhibitor than GalNAc alone |
| *Maclura pomifera* | MPA | Galactose | Galβ3GalNAc (T antigen), Galα6Glc (melibiose)  α-linked *N*-acetylgalactosamine structures |
| *Erythrina cristagalli* | ECA |  | D-galactose and D-galactosides, Galβ4GlcNAc> Lactose > GalNAc > Gal |
| *Artocarpus intergrifolia* | AIA |  | Galα6 or Gal β3GalNAc >> lactose |
| *Arachis hypogaea* | PNA |  | galactosyl (β-1,3) *N*-acetylgalactosamine, lactose |
| *Datura stramonium* | DSA | *N*-acetylglucosamine | GlcNAc β4GlcNAc oligomers, Gal β4GlcNAc |
| *Griffonia simplicifolia* | GSLII |  | Terminal *N*-acetylglucosamine (β1-2, 3 or 4  linkage) in complex N type-glycans |
| *Lycopersicon esculentum* | LEL |  | N-acetyl-β-D-glucosamine oligomers, N-acetyllactosamine oligomers |
| *Lotus tetragonolobus* | LTA | Fucose | α-linked L-fucose containing oligosaccharides, LeX but not LeA. |
| *Ulex europaeus I* | UEA-I |  | affinity for L-fucose |
| *Maackia amurensis* | MAA | Sialic acids | Gal (β-1,4) *N*-acetylglucosamine with sialic acid at the 3 position of galactose.  does not appear to bind this structure when substitution with sialic acid is on the 6 position of galactose. |
|  | MAA-II |  | sialic acid in an (α-2,3) linkage (Neu5Acα2-3Galβ1-3(NeuAcα2−6)GalNAc  . |
| *Sambucus nigra, (Elderberry bark)* | EBL (SNA) |  | sialic acid attached to terminal galactose in α-2,6  no binding to sialic acid linked to *N*-acetylgalactosamine. |
| *Phaseolus vulgaris* | PHA-L | Complex glycans | Galactose β1-4 *N*-acetylglucosamine β1-6 Mannose of tri- and  tetra-antennary |
| *Phaseolus vulgaris* | PHA-E |  | Galactose β1-4 *N*-acetylglucosamine β1-2Mannose of bi-and  tri-antennary |

^1^Adapted from [26] and manufacturer product specificity datasheet and/or website (Vector laboratories, Sigma-Aldrich)
