# Supplementary material for: Evidence for the Sialylation of PilA, the PI-2a Pilus-Associated Adhesin of Streptococcus agalactiae Strain NEM316
Source: PLoS One. 2015 Sep 25;10(9):e0138103. doi: 10.1371/journal.pone.0138103 (PMC4583379; doi:10.1371/journal.pone.0138103)
Supplement: S2 Table — (DOCX) [file pone.0138103.s007.docx]

**Table S2.** Bacterial strains and plasmids used in this study

| Strain or plasmid | Relevant properties^*^ | Source or reference |
| --- | --- | --- |
| Strains |  |  |
| *Escherichia coli* |  |  |
| XL-1 blue | *rec*A1 *end*A1 *gyr*A96 *thi-1 hsdR17 supE44 relA1 lac [F´ proAB lacIqZΔM15* Tn*10 (Tetr)].* | Stratagene |
| *Streptococcus agalactiae* |  |  |
| NEM316 | Serotype III isolated from neonate blood culture (early onset disease) | [[30](#_ENREF_30)] |
| NEM316*∆pilA* | in frame deletion of gene *gbs1478* | [[1](#_ENREF_1)] |
| NEM316*∆pilB* | in frame deletion of gene *gbs1477* | [[1](#_ENREF_1)] |
| NEM316*∆pilC* | in frame deletion of gene *gbs1474* | [[1](#_ENREF_1)] |
| NEM316*∆rogB* | in frame deletion of gene *gbs1479* | [[1](#_ENREF_1)] |
| NEM316∆*rga* | in-frame deletion of gene *gbs1530* | [[8](#_ENREF_8)] |
| NEM316-∆VWA | in-frame deletion of 180 aa of  PilA VWA domain | [[6](#_ENREF_6)] |
| NEM316-∆VWA2 | in-frame 9 aa deletion of VWA MIDAS critical residues of PilA replaced by HA epitope tag | This work |
| NEM316-N427Q | mutation replacing residue N427 into Q | This work |
| NEM316-N597Q | mutation replacing residue N597 into Q | This work |
| NEM316-N427Q-N597Q | mutation replacing both residue N427 and N597 into Q | This work |
|  |  |  |
| Plasmids |  |  |
| pG1 | Em; *oriR* pUC, *oriR^ts^* pWV01; MCS pUC18 | Trieu-Cuot and Poyart,  (unpublished vector) |
| pMSP-3545 | pAMβ1 and ColE1 replication origins, *nisRK* P*nisA*, MCS, Erm^r^ | [[27](#_ENREF_27)] |
|  |  |  |

^*^Ap and Em are resistance to ampicillin and erythromycin, respectively. MCS, multiple cloning site.
